# Biofilm Time-Kill Curves to Assess the Bactericidal Activity of Daptomycin Combinations against Biofilm-Producing Vancomycin-Resistant *Enterococcus* *faecium* and *faecalis*

**DOI:** 10.3390/antibiotics10080897

**Published:** 2021-07-23

**Authors:** Katie E. Barber, Zade Shammout, Jordan R. Smith, Razieh Kebriaei, Taylor Morrisette, Michael J. Rybak

**Affiliations:** 1 Anti-Infective Research Laboratory, School of Medicine, Eugene Applebaum College of Pharmacy and Health Sciences, Detroit, MI 48201, USA; zshammout@wayne.edu (Z.S.); jsmith5@highpoint.edu (J.R.S.); R.Kebriae@wayne.edu (R.K.); gx6737@wayne.edu (T.M.); m.rybak@wayne.edu (M.J.R.); 2 Department of Pharmacy Practice, School of Pharmacy, The University of Mississippi, 2500 North State Street, Jackson, MS 39216, USA; 3 Department of Clinical Sciences, Fred Wilson School of Pharmacy, High Point University, 1 University Parkway, High Point, NC 27268, USA; 4 Department of Internal Medicine, School of Medicine, Wayne State University, 540 E. Canfield St., Detroit, MI 48201, USA

**Keywords:** daptomycin, ceftriaxone, ampicillin, fosfomycin, rifampin, biofilm, medical device, enterococci

## Abstract

Introduction: *E. faecium* and *E. faecalis* are responsible for 13.9% of hospital-acquired infections with frequent resistance to vancomycin (82.6% of *E. faecium*, 9.5% of *E. faecalis*). Medical device infections secondary to enterococci often require combination therapy due to impaired activity against biofilm embedded cells. In vitro data demonstrate synergistic activity of daptomycin combinations. Using a novel, biofilm time-kill approach, we evaluated whether daptomycin combinations maintained synergy against biofilm-producing *E. faecium* and *E. faecalis*. Methods: Broth microdilution (BMD) and biofilm MIC (bMIC) values for daptomycin, ampicillin, ceftriaxone, fosfomycin, and rifampin were determined against biofilm-producing *E. faecium* and *E. faecalis*. Daptomycin combination bMIC values were determined in the presence of biologic concentrations of other antimicrobials. Synergy was evaluated against two *E. faecalis* (R6981, R7808) and two *E. faecium* (5938 and 8019) using a previously described biofilm time-kill method. Synergy was defined as ≥2 log10 CFU/cm^2^ reduction over the most active agent alone. Bactericidal activity was defined as ≥3 log10 CFU/cm^2^ reduction. Results: Daptomycin bMICs were 2–8-fold higher than BMD. In the presence of other antimicrobials, daptomycin bMICs were reduced ≥ two-fold in dilutions. Ceftriaxone and ampicillin demonstrated the most potent combinations with daptomycin, yielding synergy against three of four strains. Daptomycin plus rifampin was synergistic against *E. faecium* 5938 and *E. faecalis* 6981 and produced bactericidal kill. The combination of daptomycin plus fosfomycin displayed synergy solely against *E. faecalis* 6981. Conclusions: Daptomycin combinations with beta-lactams demonstrated promising synergistic activity against both *E. faecium* and *E. faecalis.* While daptomycin plus rifampin yielded bactericidal results, the effect was not seen across all organisms. These combinations warrant further evaluation to determine the optimal dose and response.

## 1. Introduction

Enterococci have been reported as one of the leading causes of all hospital-acquired infections [1]. The resistance rates in enterococci, especially to vancomycin, are increasing, leaving few alternative therapeutic options. Complicating matters further, both *Enterococcus faecium* and *E. faecalis* are capable of producing bacterial biofilm [2]. Biofilm formation encapsulates the organism, leading to an increased adherence to prosthetic material and the prevention of antimicrobial and host immune system penetration [3,4]. Additionally, these cells often display a decreased susceptibility to antimicrobials due to resistance mutations. Due to this, treatment failures are frequently observed with biofilm-associated medical device infections [5]. 

Combination therapy is typically employed to decrease the probability of antimicrobial failure. Daptomycin is likely one of the better options for combination therapy, as it provides bactericidal coverage against many vancomycin-resistant enterococci (VRE) strains and is capable of penetrating the biofilm matrix due to activity against both replicating and stationary cells [6,7]. Additionally, rifampin has the propensity to penetrate the biofilm matrix, and is utilized clinically in combination for the treatment of *Staphylococcus aureus*, making it a potentially viable option for a combination therapy for VRE [8,9]. While beta-lactams work only on actively dividing cells, synergistic activity has been observed when combined with daptomycin against biofilm-producing bacterial strains, warranting further evaluation [10,11,12,13]. Similarly, synergy has been observed between daptomycin and fosfomycin [14]. Despite both in vitro and in vivo data to support daptomycin combinations against Gram-positive organisms, it is unknown whether these combinations remain synergistic against biofilm-producing organisms. Therefore, our objective was to evaluate combinations of daptomycin and several antimicrobials, including ampicillin, ceftriaxone, fosfomycin, and rifampin, against VRE strains capable of producing bacterial biofilm.

## 2. Results

**Figure 1:**
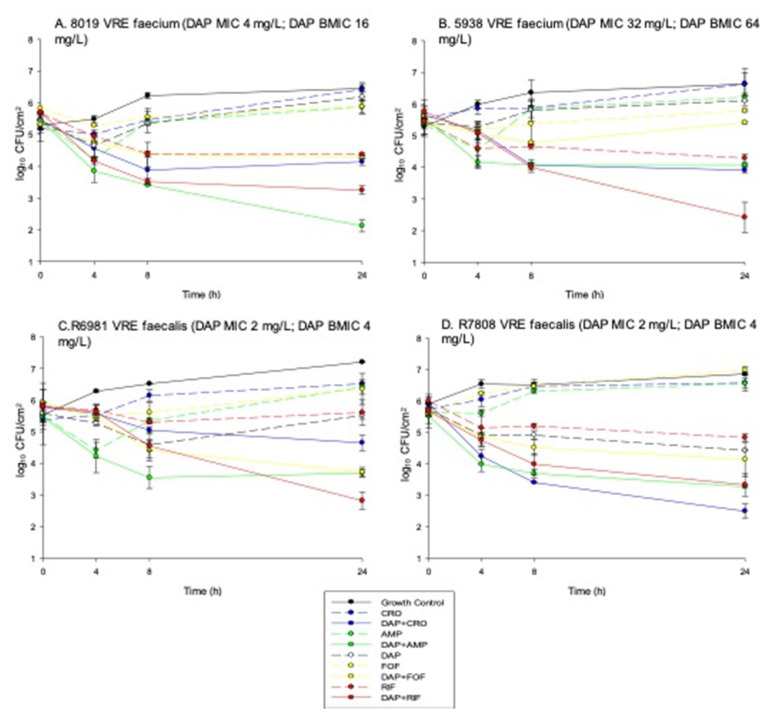
In vitro time kill curves: (**A**) *E. faecium* 8019, (**B**) *E. faecium* 5938, (**C**) *E. faecalis* 6981, (**D**) *E. faecalis* 7808 illustrating mean +/− standard deviation of CFU/cm^2^.

The baseline daptomycin MICs in the 10 enterococcal strains were as follows: three susceptible, six susceptible-dose dependent, and one resistant. Daptomycin biofilm MICs for all strains (excluding R1027) increased by ≥two-fold in dilutions from the standard broth microdilution (BMD) MIC. Seven of the ten strains displayed biofilm MICs greater than the daptomycin MIC breakpoint (>4 mg/L) for enterococci, and two of the remaining strains had bMICs at the breakpoint. In the presence of ampicillin, ceftriaxone, fosfomycin, or rifampin, daptomycin biofilm MICs decreased 16-fold, 4–8-fold, 8–32-fold, and 4–16-fold, respectively, often reducing the daptomycin bMIC to values that would be susceptible in BMD testing (Table 1). In the biofilm time-kill studies, none of the single agents displayed sustained activity, illustrated by regrowth within 24 h (Figure 1). In the daptomycin susceptible parent *E. faecium* strain (*E. faecium* 8019), a daptomycin combination with ampicillin, ceftriaxone, or rifampin displayed synergistic activity. Daptomycin plus ampicillin produced the most potent activity with a 3.0 ± 0.6 log_10_ CFU/cm^2^ reduction from baseline. Against the daptomycin non-susceptible mutant *E. faecium* strain (*E. faecium* 5938), synergistic activity with daptomycin plus either ampicillin or ceftriaxone combinations occurred, but the effect was less pronounced than the killing observed against the daptomycin susceptible parent strain. The combination of daptomycin plus rifampin produced bactericidal activity (3.4 ± 0.7 log_10_ CFU/cm^2^ reduction from baseline) against this strain, despite the other combinations demonstrating minimal activity. Daptomycin plus fosfomycin did not produce synergistic effects against either *E. faecium* strain. Against the *E. faecalis* strains, daptomycin combinations also produced synergistic effects. Against *E. faecalis* 6981, daptomycin plus rifampin, ampicillin, or fosfomycin resulted in synergy, with the rifampin combination producing bactericidal activity with a 3.0 ± 0.2 log_10_ CFU/cm^2^ reduction from baseline. Against *E. faecalis* 7808, the only combination that produced synergy was daptomycin plus ceftriaxone with a 3.4 ± 0.4 log_10_ CFU/cm^2^ reduction observed from baseline. While the other combinations did not produce a synergistic effect, 2.2 ± 0.3, 2.4 ± 0.5, and 2.3 ± 0.5 log_10_ CFU/cm^2^ reductions were observed for daptomycin combined with ampicillin, fosfomycin, and rifampin, respectively.

## 3. Discussion

Biofilm production represents a major healthcare concern. A decreased drug and host immune system exposure, a stationary growth phase, and reductions in susceptibility contribute to the poor outcomes often observed in biofilm-associated medical device infections [5]. Enterococci are a leading cause of healthcare-associated infections with vancomycin-resistance rates increasing [1]. These VRE isolates are now recognized as one of the more challenging multidrug-resistant pathogens. Their propensity for biofilm production limits therapeutic options, warranting the exploration of combination therapies that have demonstrated resistance prevention and bactericidal activity against planktonic enterococci in previous models. 

The biofilm MICs for both species of enterococci evaluated in this study displayed a 2–4-fold increase in dilutions from the standard broth microdilution MIC pending strain and antimicrobial. This increase in concentration needed to inhibit bacterial growth is consistent with the previous literature evaluating biofilm-producing organisms [10]. Additionally, we observed bactericidal activity with daptomycin plus either ampicillin, ceftriaxone, or rifampin. Unfortunately, a lack of synergy with daptomycin plus fosfomycin against three of the four biofilm-producing strains occurred. 

The activity we observed is supported by case reports of treatment success with these combinations in the clinical realm [15,16,17,18]. While the majority of data on daptomycin combinations are in staphylococci, additive, or synergistic activity has been observed with combination therapy against enterococci. Additionally, these combinations have also been studied in biofilm-producing staphylococci with success [10,19]. However, this is one of the first assessments of combination therapy against biofilm-producing enterococci.

One difference in this study compared to the previous literature is the lack of synergy observed with daptomycin plus fosfomycin. Animal data exist demonstrating the potential of this combination against enterococci [20]. As there was an inhibition of growth, a possible explanation for the lack of success with fosfomycin combinations in this study may be due to the strain selection. Additionally, it is possible that the level of biofilm produced may have made this combination less effective.

Our results demonstrate that daptomycin combination regimens, specifically combined with ampicillin, ceftriaxone, fosfomycin, or rifampin, were capable of decreasing the bacterial colony counts in several biofilm-producing enterococcal strains. While these results are promising, further research with more extensive modeling is warranted.

## 4. Materials and Methods

### 4.1. Bacterial Strains

A total of 10 clinical strains (5 *E. faecium* and 5 *E. faecalis*) with varying susceptibilities to daptomycin, including an isogenic (related) strain pair of *E. faecium*, including one daptomycin susceptible (*E. faecium* 8019) and one daptomycin non-susceptible (*E. faecium* 5938) strain, were evaluated.

### 4.2. Antimicrobials

The following antibiotics were evaluated: ampicillin, ceftriaxone, daptomycin, fosfomycin, rifampin, and vancomycin. Daptomycin (Cubist Pharmaceuticals, Lexington, MA), ampicillin, ceftriaxone, fosfomycin, rifampin, and vancomycin (Sigma Chemical Company, St. Louis, MO, USA) were purchased commercially.

### 4.3. Media

Due to the necessity of calcium for daptomycin’s antimicrobial activity, Mueller–Hinton broth II (Difco, Detroit, MI, USA) supplemented with 50 mg/L of calcium chloride and 12.5 mg/L of magnesium chloride (SMHB) was used for susceptibility testing as well as time-kill experiments. Colony counts were determined using brain heart infusion (BHI) agar plates (Difco).

### 4.4. Susceptibility Testing

Minimum inhibitory concentrations (MIC) were determined by broth microdilution per Clinical Laboratory Standards Institute (CLSI) guidelines, and biofilm MICs (bMIC) determinations were performed per the Calgary method on all VRE strains [21,22]. Daptomycin combination MICs and combination bMICs were performed in the presence of ampicillin, ceftriaxone, fosfomycin, and rifampin at 0.5 × MIC or maximum concentrations of free drug achieved in human serum utilizing standard dosing regimens [10]. All strains evaluated were proven to produce biofilm via quantification techniques utilizing well-described biofilm-forming (NRSA101 and ATCC 35556) and non-biofilm-forming (ATCC12228) strains, as previously described [23,24].

### 4.5. Biofilm Time-Kill Evaluations

A previously described methodology utilizing microwell plates to evaluate synergy against biofilm-producing organisms was utilized [10]. In brief, 3-mm polyurethane beads were placed in 1% glucose-supplemented tryptic soy broth (GSTSB), inoculated with the test organism, and incubated at 37 °C, allowing for biofilm formation. After 24 h of incubation, GSTSB was aspirated, and the beads were carefully removed via forceps and placed into wells containing Mueller–Hinton broth supplemented with 50 mg/L of calcium due to the calcium-dependent mechanism of daptomycin. Antimicrobials were added at 1× the biofilm MIC for all agents unless the biofilm MIC was greater than the free physiologic peak concentration, in which case, free physiologic peaks were used. Free peak synergistic concentrations, utilizing simulated normal human dosage regimens, were 70 mg/L for ampicillin (2 g), 25.7 mg/L for ceftriaxone (2 g), 14.7 mg/L for daptomycin (12 mg/kg), 200 mg/L for fosfomycin (4 g), and 2.1 mg/L for rifampin (300 mg). Targeted bacterial starting inoculum for all strains was 5.5–6 log_10_ CFU/cm^2^, based upon the surface area of the beads. Beads were removed with sterile forceps at 0, 4, 8, and 24 h, washed to remove adhering non-biofilm organisms, and placed into 1 mL of normal saline. Biofilm was recovered by three alternating 60-s cycles of vortexing and sonication at 20Hz Bransonic 12 Branson Ultrasonic Corporation. Recovered biofilm cells were plated on BHI agar (EasySpiral; Interscience, Worborn, MA) and incubated for 24 h at 37 °C. Synergy was defined as a ≥2 − log_10_ CFU/cm^2^ reduction over the most active agent alone. Combinations that resulted in ≥1 − log_10_ bacterial growth in comparison to the least active single agent were considered antagonistic. Single drug exposures in biofilm time-kill experiments included ampicillin, ceftriaxone, daptomycin, fosfomycin, and rifampin. Additionally, combination evaluations were performed with daptomycin plus each of the previously mentioned non-daptomycin antimicrobials.

## 5. Conclusions

Daptomycin combinations appear effective against biofilm-producing enterococci. These combinations warrant further evaluation to determine the optimal dose and response.

## Figures and Tables

**Table 1:** Standard and Biofilm Minimum Inhibitory Concentrations (mg/L).

**MIC (mg/L)**									
**Strain**	**DAP**	**AMP**	**CRO**	**FOF**	**RIF**	**DAP + AMP**	**DAP + CRO**	**DAP + FOF**	**DAP + RIF**
*E. faecalis*									
R6981	2	512	>1024	64	0.0156	0.125	0.125	0.5	0.5
R7808	2	>64	>1024	64	<0.0078	0.25	1	0.5	0.25
R6797	1	2	>64	64	2	0.25	1	0.0625	0.5
R6798	1	1	>64	64	2	0.25	0.5	<0.016	1
R6799	2	2	>64	64	2	0.25	1	0.0625	0.5
*E. faecium*									
8019	4	4	32	64	0.5	0.125	0.0625	1	0.5
5938	32	16	64	64	0.0625	0.0625	<0.031	8	8
R1026	1	32	>64	>64	<0.0156	0.5	1	0.125	0.25
R1027	2	32	>64	64	4	1	1	0.25	0.25
R1028	4	32	>64	>64	32	1	1	0.5	0.015
**Biofilm MIC (mg/L)**									
**Strain**	**DAP**	**AMP**	**CRO**	**FOF**	**RIF**	**DAP + AMP**	**DAP + CRO**	**DAP + FOF**	**DAP + RIF**
*E. faecalis*									
R6981	4	>64	>64	>64	0.03125	0.25	0.5	0.25	0.25
R7808	4	>64	>64	>64	0.0156	0.25	1	0.5	0.5
R6797	8	>64	>64	>64	2	1	4	1	8
R6798	8	>64	>64	>64	2	2	4	2	8
R6799	8	>64	>64	>64	1	2	4	1	8
*E. faecium*									
8019	16	>64	>64	>64	1	1	2	0.5	2
5938	64	>64	>64	>64	0.25	4	8	8	16
R1026	8	64	>64	32	0.0156	1	1	1	0.25
R1027	2	64	>64	32	<0.031	1	1	1	0.25
R1028	8	>64	>64	>64	>64	1	1	1	0.5

DAP: daptomycin; AMP: ampicillin; CRO: ceftriaxone; FOF: fosfomycin; RIF: rifampin.

## Data Availability

The data presented in this study are available on request from the corresponding author.
